# Enhanced Plasticity of Human Evoked Potentials by Visual Noise During the Intervention of Steady-State Stimulation Based Brain-Computer Interface

**DOI:** 10.3389/fnbot.2018.00082

**Published:** 2018-11-29

**Authors:** Jun Xie, Guanghua Xu, Xingang Zhao, Min Li, Jing Wang, Chengcheng Han, Xingliang Han

**Affiliations:** ^1^School of Mechanical Engineering, Xi'an Jiaotong University, Xi'an, China; ^2^State Key Laboratory of Robotics, Shenyang Institute of Automation, Chinese Academy of Sciences, Shenyang, China; ^3^State Key Laboratory for Manufacturing Systems Engineering, Xi'an Jiaotong University, Xi'an, China

**Keywords:** brain-computer interface (BCI), motion-reversal stimulation, plasticity, visual evoked potential (VEP), visual noise

## Abstract

Neuroplasticity, also known as brain plasticity, is an inclusive term that covers the permanent changes in the brain during the course of an individual's life, and neuroplasticity can be broadly defined as the changes in function or structure of the brain in response to the external and/or internal influences. Long-term potentiation (LTP), a well-characterized form of functional synaptic plasticity, could be influenced by rapid-frequency stimulation (or “tetanus”) within *in vivo* human sensory pathways. Also, stochastic resonance (SR) has brought new insight into the field of visual processing for the study of neuroplasticity. In the present study, a brain-computer interface (BCI) intervention based on rapid and repetitive motion-reversal visual stimulation (i.e., a “tetanizing” stimulation) associated with spatiotemporal visual noise was implemented. The goal was to explore the possibility that the induction of LTP-like plasticity in the visual cortex may be enhanced by the SR formalism via changes in the amplitude of visual evoked potentials (VEPs) measured non-invasively from the scalp of healthy subjects. Changes in the absolute amplitude of P1 and N1 components of the transient VEPs during the initial presentation of the steady-state stimulation were used to evaluate the LTP-like plasticity between the non-noise and noise-tagged BCI interventions. We have shown that after adding a moderate visual noise to the rapid-frequency visual stimulation, the degree of the N1 negativity was potentiated following an ~40-min noise-tagged visual tetani. This finding demonstrated that the SR mechanism could enhance the plasticity-like changes in the human visual cortex.

## Introduction

Neuroplasticity, also known as brain plasticity and neural plasticity, is an inclusive term that covers the permanent changes in the brain during the life of an individual. Neuroplasticity can be broadly defined as the changes in function or structure of the brain in response to the external and/or internal influences. As a ubiquitous form of functional brain plasticity, long-term synaptic plasticity modulates the network function with the strengthening of synaptic transmission from several minutes to many months in many brain areas (Abraham, 2003). Here, long-term potentiation (LTP) refers to an increased activity-dependent reorganization of the synaptic networks after persistent stimulation (Borroni et al., 2000; Cooke and Bliss, 2006), which fulfills many of the criteria for learning and memory (Bliss and Collingridge, 1993; Kandel, 2001).

LTP was often induced by applying a rapid-frequency stimulation (or “tetanus”) to an afferent pathway. This tetanus ensures the concurrent pre- and post-synaptic depolarization, and increases excitability of the dendritic spines of the post-synaptic neurons, leading to a lasting enhancement of synaptic strength (Bliss and Lømo, 1973). Repetitive stimulation of the brain can produce long-lasting increases in excitatory drive (Cooke and Bliss, 2006), and potentially provide therapies for neurological disorders that are derived from the output reduction of particular brain regions, such as in Parkinson's-like motor deficits (Filipović et al., 2010), depression (Huang et al., 2005), epilepsy (Tergau et al., 1999), and hyperalgesia (Arendt-Nielsen et al., 2000). Most recently, the LTP has been extensively studied at the microscopic level in individual neurons, e.g., in experiments on rodent brain slices (Van Praag et al., 1999; Huber et al., 2002) and human hippocampus *in vitro* (Beck et al., 2000). However, due to the invasive character of the above research, only a few *in vivo* human studies have been implemented thus far, limiting our understanding of neuroplasticity in the living human brain.

Sensory experience is important in the formation of neuronal connections during the critical early period of an individual's life (Hubel and Wiesel, 1970). Although this point of view still holds true, many researchers have challenged that notion and indicated that the visual system is continuously regulated by environmental changes and that its neuroplasticity is altered even in adulthood (Frégnac et al., 1988; Karni and Sagi, 1991; Sale et al., 2011). Visual neuroplasticity is mainly induced by the sustained resonance of brain neurons, triggered by various repetitive rapid-frequency visual stimulation paradigms (Stefan et al., 2000). Several animal and human studies have reported that a rapid presentation of visual tetani could produce repeated or persistent firing of neurons, and thus a long-lived strengthening of post-synaptic cells' connections and the induction of LTP-like changes could be observed. Moreover, methods are available to assess the synaptic transmission and corresponding plasticity in the living animal and human brain by examining the evoked responses elicited by “tetanizing” stimulation. Studies in laboratory animals had demonstrated that when rodents were exposed to repeated presentation of visual stimuli, the visual evoked response was enhanced. In the human visual cortex, one of the early components of the visual evoked potentials (VEPs), i.e., the N1 component, can be potentiated after rapid and repetitive visual stimulation (Teyler et al., 2005; Normann et al., 2007; Ross et al., 2008; Cooke and Bear, 2012) and is reminiscent of synaptic LTP induced by tetanic stimulation (Karmarkar and Dan, 2006). The aforementioned evidence from human and rodent studies supports the possibility of inducing LTP-like alterations with a non-invasive visual tetanus. It also indicates that the selective alternation of a single component of the VEPs, which is derived from the summation of electrical fields generated from a large number of neurons, represents a form of synaptic plasticity (Clapp et al., 2012).

Modifications of neural circuits not only depend on the pattern of the sensory inputs but also on the network of neurons that receives them (Karmarkar and Dan, 2006). By modulating the excitability of neuronal network, random noise stimulation could optimize the effect of behavioral training with measurable brain changes, which may ultimately lead to neuroplasticity (Fertonani et al., 2011). Indeed, recent empirical studies suggested that transcranial random noise stimulation (tRNS), i.e., a non-invasive brain stimulation (NIBS) method using alternating current at random intensities and frequencies, could be related to the enhancement of neural plasticity due to the strengthening of synaptic transmission between neurons via the stochastic resonance (SR) mechanism (Joos et al., 2015). This method could boost synaptic signals in the motor cortex and elicit functional neuroplastic changes in stroke survivors (Martínez et al., 2007; Terney et al., 2008). However, studies of visual neuroplasticity via the SR formalism are lacking.

Therefore, the purpose of the present work was to use an established non-invasive measure of LTP to assess the impact of visual noise on neuroplasticity. A brain-computer interface (BCI) intervention based on rapid and repetitive steady-state motion-reversal visual stimulation associated with spatiotemporal visual noise was conducted to explore the influence of SR formalism on the visual neuroplasticity. The SR formalism achieves the benefits of visual noise on the foundation of periodic visual stimulation. Because VEPs represent electroencephalographic (EEG) potentials correlated with post-synaptic neuronal activity which can be considered as a marker to provide plasticity-like information of synaptic activity and cortical excitability, we used this non-invasive potentiation of VEPs as an accessible method to investigate the visual neuroplasticity in healthy subjects. We focused on the very early P1 and N1 components of VEP potentials, as they are less likely to be influenced by attention or complex cognitive processes and may characterize synaptic transmission (Hillyard and Anllo-Vento, 1998; Normann et al., 2007). Consequently, late VEP potentials (such as the P300 component), which are modulated by more complex cognitive processing, were not the concern of this study.

## Materials and methods

### Subjects

Four female and seven male subjects (aged 23–29 years old) participated in this study. They were all healthy right-handed graduate students from Xi'an Jiaotong University (Shaanxi, China) and had normal or corrected-to-normal vision. All subjects had experimented with the steady-state visual evoked potential (SSVEP)-based BCI before but were naive to the steady-state motion visual evoked potential (SSMVEP)-based BCI paradigm masked with visual noise. The experiment was undertaken in accordance with the recommendations of the Declaration of Helsinki, and the study was approved by the Institutional Review Board of Xi'an Jiaotong University. All subjects provided informed written consent before the start of the experiments.

### EEG recordings

EEG signals were recorded using a single active gold-cup scalp electrode placed over the primary visual cortex of the occipital head (Oz) where maximum VEP effects have been identified (Normann et al., 2007). The ground electrode was placed on the forehead (Fpz) site, and the reference electrode was attached to a unilateral earlobe. The EEG signals were acquired with a g.USBamp acquisition device (g.tec Medical Engineering GmbH Schiedlberg, Austria) at a sampling rate of 1,200 Hz to ensure the encompassment of the stimulation frequency's single cycles to alleviate the spectral leakage (Bach and Meigen, 1999; Wilson and Palaniappan, 2011). An analog band-pass filter (BPF) with a pass-band of 2–100 Hz and a notch filter with a stop-band of 48–52 Hz were applied to remove, respectively, electrophysiological and motion artifacts and mains interference. The impedance of all electrodes was kept to be < 5 K ohms during the experiments.

### Stimulation design

A gamma-corrected 22-inch Dell LCD screen simultaneously presented the subject with three motion-reversal stimulation targets at a resolution of 1,024 × 768 pixels. The motion reversal stimulation procedure was designed according to our earlier studies (Xie et al., 2012, 2014); each target was constructed from a white motion ring with its width being constant during the entire motion process and equal to half the radius of the circular region of the motion ring (maximum Michelson contrast: 98.8%). In addition, according to the previous study demonstrating that stimuli of size beyond 3.8° of visual angle would make the elicited VEPs reach a plateau (Ng et al., 2012), the diameter of the circular region was selected to be 4.8° in the present study. The motion-reversal frequencies of the three targets were selected as unique, constant, and harmonically unrelated frequencies. Thus, 15, 12, and 8.6 Hz were selected in accordance with the screen refresh rate of 60 Hz. These three tetanic-stimulation frequencies were also chosen because that they remain below the critical flicker fusion rate of typically 50–60 Hz (Teyler et al., 2005; Sakurada et al., 2015). The arrangement of the three targets formed an equilateral triangle, and the frequencies of 15, 12, and 8.6 Hz were arranged to the lower right, lower left and the upper corner, respectively. The spatial proximity from the center of the screen to each target was 7.2°. For the application of noise-tagged BCI intervention, a moderate level of visual noise was masked on the visual target. The visual noise had a spatial-temporal characteristic of noise spots changing dynamically with time. Specifically, each noise spot was a square region containing a 5 min visual angle with brightness changes following the Gaussian distribution with mean of 128 and standard deviation (SD) of 40 of gray-scale level. The noise spots spanned the entire screen as background stimulation to mask the targets and were updated in 1/60 s in accordance with 60 Hz refresh rate. In the pause between each trial, a spatially homogeneous gray background with the same average luminance as the motion-reversal pattern was presented. The presentation of the novel SSMVEP (i.e., a specific type of SSVEP) paradigm based on steady-state motion-reversal visual stimulation was introduced into the spatial selective attention-based BCI system with the programming using the Psychophysics Toolbox (http://psychtoolbox.org/) (Brainard, 1997; Pelli, 1997), which would alleviate the mental load and fatigue effects according to our previous study (Xie et al., 2016). In the entire experiment, each subject was 70 cm from the screen, and the center of the screen was at the horizontal position of the subject's eye to achieve selective attention to each stimulus target.

### Online experimental setup and target detection procedure

**Figure 1:**
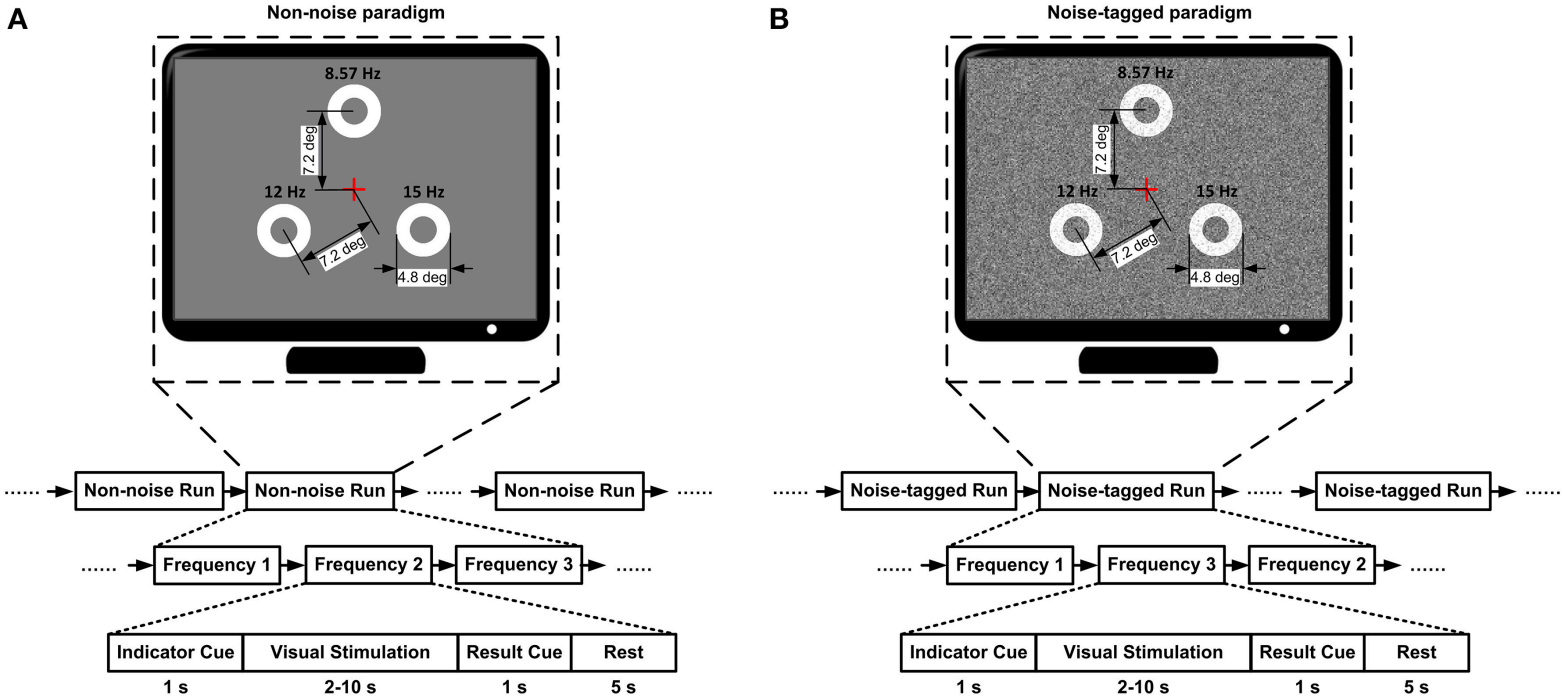
The overall BCI experimental protocol. **(A)** The non-noise BCI session. **(B)** The noise-tagged BCI session. For both the non-noise and noise-tagged sessions, the three targets were masked by noise spots based on background stimulation with standard deviations 0 and 40 of gray-scale level, respectively. Each run contained 15 trials. Each trial started with a visual cue that appeared for 1 s above a specific target to instruct subjects to pay attention to that target. Following the cue offset, three motion-reversal targets were simultaneously displayed on the screen for 2–10 s. Subjects were asked to maintain attention and watch closely the indicated target. Once the target was identified, another visual cue was presented in the center of the screen for 1 s to display the result and the trial was terminated. After the stimulus was offset, a homogenous gray screen was presented for 5 s before the next trial began.

The overall BCI experimental protocol is illustrated in Figure 1. The online BCI interventions were divided into two types of non-noise and noise-tagged tasks, where each task was performed in a separate session and the non-noise task served as the control group. Subjects were instructed to fixate at every 15, 12, and 8.6 Hz stimulation in random order (see Figure 1). The random sequence was performed to avoid adaptation and habituation of lone-term stimulation that could potentially affect assessment of SR effect (Bergholz et al., 2008). Each task involved 7–8 runs for every subject and each run included 15 trials. The BCI interventions were performed in a time-variable manner, so that the period of trials could last between 2–10 s with 0.5-s increment, depending on the brain response detection. This type of design would motivate the BCI intervention to close the brain training loop that could emphasize neurofeedback-induced changes in neuroplasticity (Sitaram et al., 2017). In the time course of each trial, a 1-s cue appeared first above a specific target, which would instruct subjects to attend to that target. Once that target was identified, another 1-s cue was presented in the center of the screen to display the recognition result and the trial was terminated. The inter-trial interval was fixed to 5 s. Resting periods of several minutes were reserved between runs at the discretion of subjects; the subjects were also advised to close their eyes for a moment before they continued the task to allow any visual after-effects to disappear. For each subject, each experimental session usually lasted ~40 min. Because subjects were not allowed to blink eyes or move their bodies during each run, the horizontal or vertical electrooculogram (EOG) signals were not recorded and, therefore, trials contaminated by few artifacts were not excluded.

For each trial data, the *GT*${}_{circ}^{2}$ statistic method (Xie et al., 2017) was used to determine the presence of SSMVEP at each stimulating frequency and its sub-harmonic. Three rectangular windows containing three cycles of three stimulating frequencies (840 sampling numbers of 8.6-Hz stimulation, 600 sampling numbers of 12-Hz stimulation, and 480 sampling numbers of 15-Hz stimulation) were separately slid over each trial with one-cycle overlap (280 sampling numbers of 8.6-Hz stimulation, 200 sampling numbers of 12-Hz stimulation, and 160 sampling numbers of 15-Hz stimulation), according to the sampling rate of 1,200 Hz. The acquired data samples were transformed to the FFT, and complex Fourier vectors involving each stimulating frequency and its sub-harmonic were obtained in four-dimensional form. If the length of trials was not expressed as an integer periods of corresponding stimulating frequency, the remainders were truncated to avoid the spectral leakage. The *GT*${}_{circ}^{2}$ provides a hypotheses-test method to determine whether the acquired FFT vectors contain only pure random noise or they include periodic components at a given confidence level. In this study, the confidence level was set at 0.99. The stimulus with the maximal confidence probability which also exceeded the given confidence level was considered to be the desired target. The period of the motion-reversal procedure increased until the desired target was recognized as the same in two successive recognitions with the interval of 0.5 s. If *GT*${}_{circ}^{2}$results could not achieve the predefined confidence level for any of the three targets beyond 10 s, this trial would be terminated without any cue.

### Data extraction

To investigate neuroplasticity in both the non-noise and noise-tagged BCI interventions, the changes in VEPs were evaluated. Transient VEPs were evoked and collected from the transient state of SSMVEP signals during the initial presentation period of each trial's steady-state motion-reversal visual stimulation. After completion of data collection, EEG data were segmented with respect to event markers into 350-ms epochs (including a 100-ms pre-stimulus baseline). Since the reported effects on synaptic plasticity are often weak and may be highly variable among individuals, for each subject the magnitudes of EEG epochs were calculated by subtracting the pre-tetanus baseline from the post-tetanus amplitudes to minimize the inter-subject variability (Huang et al., 2005). The individual epochs of the 11 subjects from which the VEP data was derived were pooled according to the stimulus condition, and then averaged across runs of repeated presentation of the same stimulus, yielding averaged VEPs for each non-noise or noise-tagged condition. The N1 and P1 peaks were identified using the averaged data. The absolute amplitude of the P1 component was determined as the first positive peak of the transient VEPs, and the absolute amplitude of the N1 component was determined as the negative peak between P1 and P2 components. The P1 and N1 amplitudes measured from the experimental epochs of the first single runs of all 11 subjects across all three stimulation frequencies were extracted to represent the early post-tetanus periods. And the P1 and N1 amplitudes measured from the experimental epochs of the last single runs of all 11 subjects across all three stimulation frequencies were extracted to represent the late post-tetanus periods.

### Statistical analysis

Using these parameters, the N1 and P1 peak amplitudes from each subject were calculated for statistical analysis. The amplitude difference was analyzed for each single component of the VEPs across the non-noise and noise-tagged BCI conditions by a one-way analysis of variance (ANOVA) statistic, which would take into account the within- and between-group variances for the factor of inter-subject variability. The level of statistical significance was set at *p* < 0.05. Results are expressed as mean ± SD.

## Results

By using visual tetani, the strength of the scalp EEG responses was modified via prolonged activation of the visual pathway to inspect the induction of LTP-like plasticity reflected as changes in the amplitude of VEPs. The transient response usually emerges when the ongoing, random-phase activity in the visual cortex is locked to the onset of a visual stimulus. In addition, the steady-state response appears to originate from the summation of the closely overlapped transient responses. Consequently, these two phenomena have often been treated as arising from the same neuronal sources (Galambos et al., 1981; Bohórquez and Özdamar, 2008), where the VEP obtained from the initial transient period of the steady-state stimulus resemble a motion-onset VEP elicited by transient motion stimulation for neuroplasticity evaluation.

### The target detection performance

Across subjects, grand-averaged online detection accuracies significantly increased by 12% (one-way ANOVA: *F* = 22.25, *p* < 0.001) at the noise-tagged condition (80.37% ± 10.20) as compared to the non-noise condition (68.33% ± 11.42). Conversely, grand-averaged detection time under the noise-tagged condition was significantly shorter than under the non-noise condition (noise-tagged, 2.81 ± 0.61 s vs. non-noise, 3.21 ± 1.15 s; unbalanced one-way ANOVA: *F* = 32.99, *p* < 0.001). The high accuracy achieved with the detection time close to the predefined minimal value of 2.5 s at the noise-tagged condition implied that the neurofeedback-mediated online BCI with visual noise could shorten the time lag between the presentations of stimulation and the induction of response. This would more effectively close the brain training loop, potentially leading to the strengthening of neuroplasticity in humans.

### The intra- and inter- subject comparisons of P1 and N1 latencies

Using the averaged data from the 15, 12, and 8.6-Hz stimulation frequencies, the beginning of the steady-state response, which was evoked by the initial presentation of the steady-state stimulation, was dominated by strong positivity and negativity before a continuous oscillation emerged. Then, the oscillation would sustain during the remaining steady-state phase. The N1 component was a negative waveform peaking at ~160–190 ms after the stimulus onset. The earlier positive P1 component occurred around 100 ms post-stimulus. For Subject S1, a positive peak at 116.1 ± 10.7 ms (P1 component) and a negative peak at 167.7 ± 11.0 ms (N1 component) could be regularly identified during the initial presentation period of the non-noise visual stimulation. Similarly, a positive peak at 115.0 ± 9.8 ms (P1 component) and a negative peak at 163.5 ± 12.5 ms (N1 component) could be regularly identified during the initial presentation period of the noise-tagged visual stimulation, with small variance in latencies between the non-noise and the noise-tagged conditions (*F* = 0.12, *p* = 0.736 for the P1 component; *F* = 1.12, *p* = 0.297 for the N1 component). The same trend of variation could also be found from Subject S2 to Subject S11 (*p* > 0.05 for P1 positivity and N1 negativity comparisons). Specific mean values and SD of P1 and N1 latency variations under the non-noise and the noise-tagged conditions for each subject are summarized in Table 1. These results indicated that for each subject, the latency variance between the non-noise and the noise-tagged conditions was significantly small. Thus, the latencies under these two conditions could be combined to evaluate the inter-subject variability.

**Table 1:** Statistical summary of the P1 and N1 latency variations in both the non-noise and noise-tagged conditions for individual subjects.

**Subjects**	**P1 Latency (ms)**				**N1 Latency (ms)**			
	**Non-noise**	**Noise-tagged**	**ANOVA**		**Non-noise**	**Noise-tagged**	**ANOVA**	
	**Mean (SD)**	**Mean (SD)**	***F***	***p***	**Mean (SD)**	**Mean (SD)**	***F***	***p***
**S1**	116.1 (10.7)	115.0 (9.8)	0.12	0.736	167.7 (11.0)	163.5 (12.5)	1.12	0.297
**S2**	119.8 (17.0)	121.9 (13.6)	0.16	0.688	175.5 (17.6)	175.1 (11.4)	0.01	0.941
**S3**	115.6 (10.9)	117.4 (10.5)	0.28	0.601	171.4 (13.7)	171.4 (13.7)	< 0.01	0.985
**S4**	118.4 (12.9)	118.9 (13.9)	0.01	0.909	175.4 (12.3)	174.0 (12.5)	0.14	0.711
**S5**	112.4 (12.9)	111.7 (9.5)	0.04	0.852	173.2 (15.3)	165.8 (10.6)	2.36	0.136
**S6**	111.9 (14.2)	113.2 (10.7)	0.12	0.731	171.8 (11.5)	169.5 (9.7)	0.59	0.445
**S7**	115.6 (13.7)	120.6 (15.0)	0.89	0.353	181.2 (11.6)	181.6 (14.6)	< 0.01	0.945
**S8**	125.4 (15.0)	123.0 (14.1)	0.25	0.623	179.8 (13.0)	180.0 (19.3)	< 0.01	0.960
**S9**	119.7 (22.6)	119.1 (15.8)	< 0.01	0.945	171.2 (17.4)	179.9 (19.0)	1.03	0.326
**S10**	121.2 (17.2)	126.0 (15.9)	0.50	0.486	184.2 (14.4)	179.7 (17.0)	0.48	0.497
**S11**	112.1 (17.8)	121.8 (13.4)	2.28	0.145	170.3 (6.3)	177.0 (12.3)	2.80	0.108

*Non-noise Mean, mean values of the P1 and N1 latencies in the multi-run non-noise data; Noise-tagged Mean, mean values of the P1 and N1 latencies in the multi-run noise-tagged data; SD, standard deviation*.

One-way ANOVA with Bonferroni-corrected multiple comparisons revealed that there was a small, statistically non-significant, inter-subject variability in latencies for the P1 component (*F* = 1.58, *p* = 0.155). For latencies of the N1 component, similar results could also be found (*F* = 1.15, *p* = 0.335). These indicated that the grand averaging of the waveforms observed in different subjects could be performed.

### The plastic modulation of VEP amplitudes by visual noise

**Figure 2:**
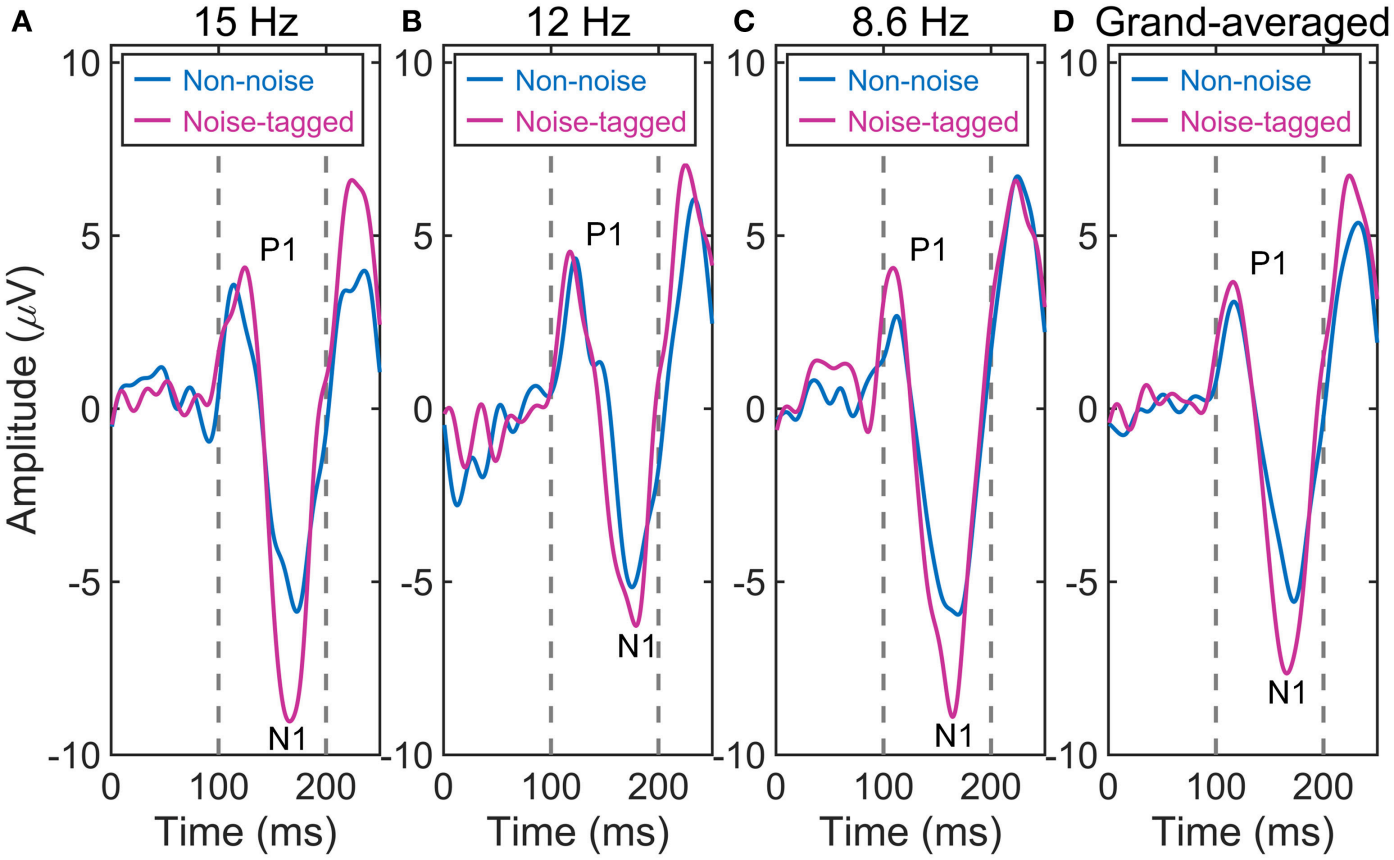
The comparison of the averaged VEP waveforms between non-noise and noise-tagged conditions across the eleven subjects. **(A)** The averaged VEPs from the stimulation frequency of 15 Hz. **(B)** The averaged VEPs from the stimulation frequency of 12 Hz. **(C)** The averaged VEPs from the stimulation frequency of 8.6 Hz. **(D)** The grand-averaged VEPs from stimulation frequencies of 15, 12, and 8.6 Hz.

We tested whether the plastic modulation of the VEPs was dependent on different noise conditions. Thus, the amplitude of every single component of the VEPs was separately analyzed with a one-way ANOVA to detect any significant change over time. Figure 2 shows that the averaged VEPs, recorded in 11 subjects over the occipital cortex and driven by noise-tagged motion-reversing visual stimuli, undergo a striking increase in amplitude across runs of repeated presentation of the same stimulus compared with the non-noise condition. For the 15 and 12-Hz stimulation frequencies, the amplitude of P1 positivity was increased, albeit non-significantly, after the noise-tagged BCI intervention compared with the non-noise “tetanizing” stimulation. This increase was seen for both 15-Hz stimulation frequency (noise-tagged: 5.4 ± 2.1 μV vs. non-noise: 4.8 ± 2.6 μV; *F* = 1.63, *p* = 0.205) and 12-Hz stimulation frequency (noise-tagged: 5.2 ± 2.5 μV vs. non-noise: 4.9 ± 2.1 μV; *F* = 0.33, *p* = 0.569). In contrast, the amplitude of N1 negativity was significantly potentiated after the noise-tagged BCI intervention compared with the non-noise condition in both 15-Hz stimulation frequency (noise-tagged: −7.7 ± 3.6 μV vs. non-noise: −6.4 ± 2.9 μV; *F* = 4.46, *p* = 0.037) and 12-Hz stimulation frequency (noise-tagged: −7.2 ± 3.3 μV vs. non-noise: −6.1 ± 2.1 μV; *F* = 4.25, *p* = 0.042). For the 8.6-Hz stimulation frequency, the amplitudes of P1 positivity and N1 negativity were both significantly potentiated after the noise-tagged BCI intervention compared with the non-noise condition. The P1 amplitude ranged from 3.6 ± 1.9 μV in the non-noise condition to 5.3 ± 2.9 μV in the noise-tagged condition (*F* = 12.93, *p* < 0.001), while the N1 amplitude ranged from −7.3 ± 2.8 μV in the non-noise condition to −8.9 ± 3.7 μV in the noise-tagged condition (*F* = 6.00, *p* = 0.016). The grand-averaged VEPs calculated from the 15, 12, and 8.6-Hz stimulation frequencies revealed that the amplitude of the P1 component increased significantly by 18% after the noise-tagged BCI intervention compared with the non-noise condition (noise-tagged: 5.3 ± 2.5 μV vs. non-noise: 4.5 ± 2.3 μV; *F* = 9.91, *p* = 0.002), while the N1 negativity was also significantly increased by 20% after the noise-tagged BCI intervention compared with the non-noise condition (noise-tagged: −7.9 ± 3.6 μV vs. non-noise: −6.6 ± 2.6 μV; *F* = 14.28, *p* < 0.001).

### The comparison of VEP amplitudes between the early and late post-tetanus periods

**Figure 3:**
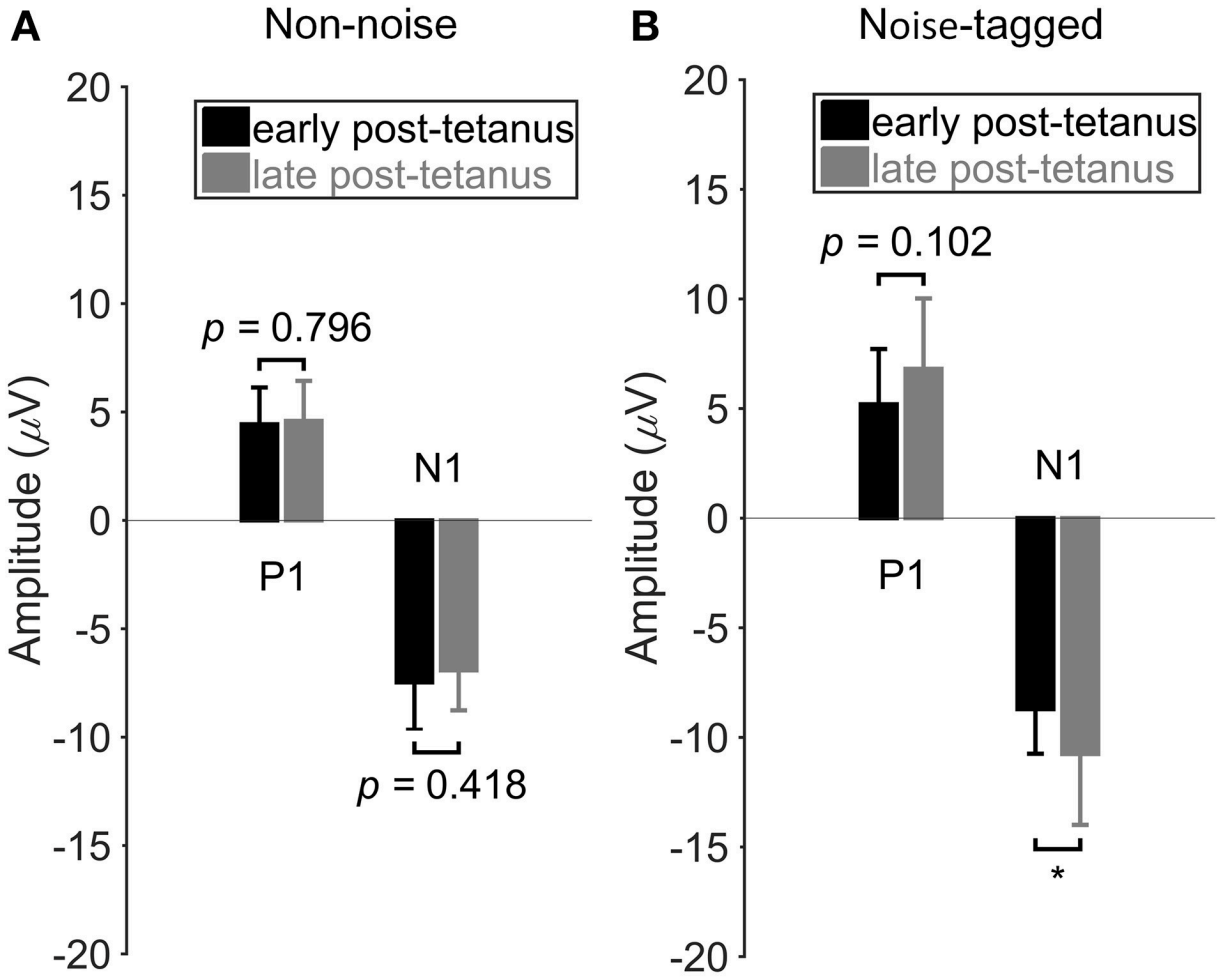
The comparison of VEP amplitudes between the early and late post-tetanus periods in either the non-noise or the noise-tagged condition across subjects. **(A)** The mean values and SD of the P1 positivity and N1 negativity in the early and late post-tetanus periods were calculated in the non-noise condition across the 11 subjects. **(B)** The mean values and SD of the P1 positivity and N1 negativity in the early and late post-tetanus periods were calculated in the noise-tagged condition across the 11 subjects. All statistics were assessed by one-way ANOVA, *p < 0.05 between the early and late post-tetanus periods in either the non-noise or the noise-tagged BCI intervention.

It was hypothesized that the noise-tagged “tetanizing” stimulation-based BCI intervention would show a greater potentiation of VEPs than the non-noise condition, as evidenced by the greater increase in N1 negativity after a prolonged (e.g., ~40 min in the present study) task. Therefore, more sophisticated statistical analysis of the P1 and N1 amplitude differences between the early post-tetanus periods and the late post-tetanus periods was performed under the non-noise and noise-tagged BCI interventions across the 11 subjects (Figure 3).

Figure 3 shows the P1 and N1 component differences between the early and late post-tetanus periods for both the non-noise and noise-tagged BCI interventions across the 11 subjects. As expected, for the noise-tagged BCI intervention, the N1 component was significantly potentiated after the “tetanizing” stimulation (124%; *F* = 5.16, *p* = 0.030) during the late post-tetanus period compared with the corresponding early post-tetanus period (late post-tetanus: −10.8 ± 3.2 μV vs. early post-tetanus: −8.7 ± 2.0 μV). There was no significant potentiation but rather a lowering of the amplitude of N1 negativity in the late post-tetanus period compared with the early post-tetanus period within the non-noise BCI intervention (late post-tetanus: −6.9 ± 1.9 μV vs. early post-tetanus: −7.5 ± 2.2 μV; *F* = 0.67, *p* = 0.418). The P1 component also exhibited an increased amplitude, i.e., a potentiation effect, in the late post-tetanus VEPs (6.8 ± 3.2 μV) compared with the corresponding P1 component of early post-tetanus VEPs (5.2 ± 2.5 μV) in the noise-tagged BCI intervention. However, this change did not reach statistical significance (*F* = 2.82, *p* = 0.102). In contrast, no significant or observable modulation of P1 amplitudes could be detected between the early and late post-tetanus periods within the non-noise BCI intervention (late post-tetanus: 4.6 ± 1.9 μV vs. early post-tetanus: 4.4 ± 1.7 μV; *F* = 0.07, *p* = 0.796).

### The extent of VEP amplitudes is enhanced by visual noise in the late post-tetanus periods

**Figure 4:**
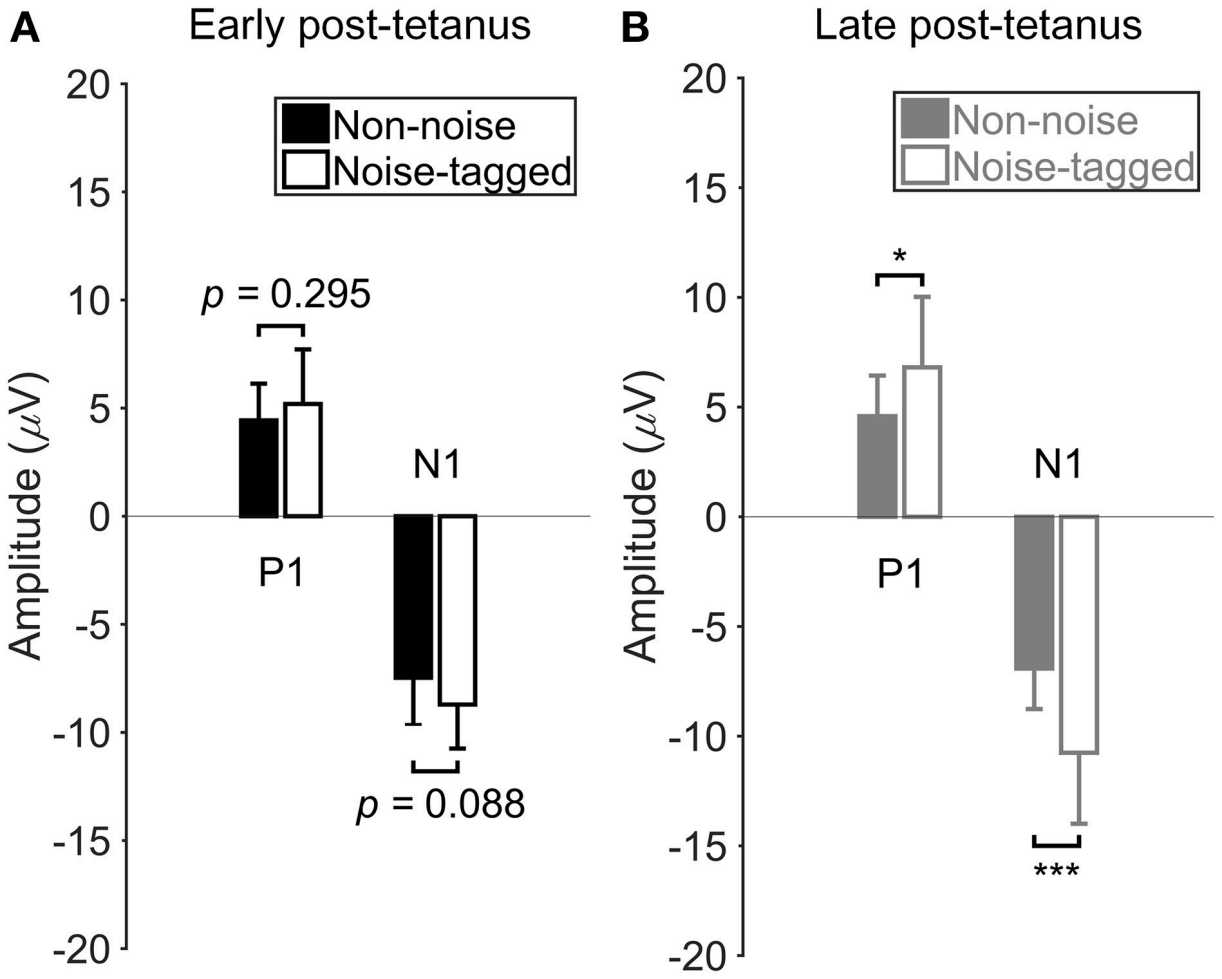
The comparison of VEP amplitudes between the non-noise and noise-tagged conditions in either the early or the late post-tetanus periods across subjects. **(A)** The mean values and SD of the P1 positivity and N1 negativity in the early post-tetanus periods were calculated in both the non-noise and noise-tagged conditions across the 11 subjects. **(B)** The mean values and SD of the P1 positivity and N1 negativity in the late post-tetanus periods were calculated in both the non-noise and noise-tagged conditions across the 11 subjects. All statistics were assessed by one-way ANOVA, *p < 0.05 between the non-noise and noise-tagged BCI interventions, ***p < 0.001 between the non-noise and noise-tagged BCI interventions.

Figure 4 illustrating the above-mentioned plasticity potentiation effect of visual noise after an ~40-min visual stimulation, shows no significant or observable differences in the N1 negativity and P1 positivity in the early post-tetanus periods between the non-noise and noise-tagged BCI interventions (*F* = 3.09, *p* = 0.088 for the N1 negativity comparisons; *F* = 1.13, *p* = 0.295 for the P1 positivity comparisons). In contrast, the N1 component of the late post-tetanus VEPs measured in the noise-tagged BCI intervention yielded a significantly higher potentiation effect in amplitude compared with the corresponding N1 component in the late post-tetanus period of the non-noise condition (*F* = 19.12, *p* < 0.001). Moreover, the noise-tagged BCI intervention also resulted in significantly higher P1 amplitude in the late post-tetanus period than the corresponding P1 component of the late post-tetanus period of the non-noise condition (*F* = 6.47, *p* = 0.016). These results indicated that the noise-tagged BCI intervention provided a superior enhancement of the potentiation effect rather than the conventional non-noise BCI paradigm during prolonged usage.

## Discussion

In this study, we used one of the fundamental functions of BCIs, i.e., neuromodulation (Nudo et al., 1996), to induce neuroplasticity in healthy humans. Our findings provide the first *in vivo* evidence that the noninvasive action of rapid and repetitive “tetanizing” stimulation associated with a moderate spatiotemporal visual noise could exert noise-enhanced plasticity-like changes of evoked potentials in the human visual cortex. A steady-state motion-reversal visual stimulation was delivered at 15, 12, and 8.6 Hz and combined with the application of visual noise for ~40 min. After exposure to the prolonged “tetanizing” stimulation protocol via the SR formalism, an overall potentiation of the early components of subsequent VEPs were observed compared with the non-noise BCI intervention. The steady-state component was also affected by the SR-based stimulation and provided higher accuracy and shorter reaction time to shorten the time lag between stimulation presentation and brain reaction, which would more effectively close the brain training loop and be the reason that the neuroplasticity studied in the present work was strengthened by visual noise. Specifically, the N1 component seemed to be more prone to potentiation, as uncovered in the experiments of Teyler et al. (2005). Here we also demonstrated significant plasticity-like changes in healthy subjects as evidenced by a significantly potentiated N1 negativity in the late post-tetanus period (i.e., measured after ~40 min of stimulation) as compared with the early post-tetanus period in the noise-tagged condition. The amplitude of the P1 component was also increased but did not change significantly. Regarding the non-noise BCI intervention, a non-significant opposite phenomenon, showing lower N1 negativity, appeared in the late post-tetanus period compared with the early post-tetanus period. The clear reduction of the VEP amplitudes after the motion after-effect may be due to a motion adaptation (Heinrich and Bach, 2003). The potential existence of an adaptation effect may be due to the fact that, in the case of radial motion stimulation, we used two opposite motion directions of expansion and contraction, which was proven to significantly reduce but not fully eliminate the direction-specific adaptation (Heinrich and Bach, 2003). It is also worth noting that there was a significant difference in the “late period” plasticity-like modulation effect between the non-noise and the noise-tagged conditions. Specifically, the “tetanizing” stimulation with visual noise resulted in a significantly greater increase in N1 negativity in the late post-tetanus period than the corresponding N1 component of the late post-tetanus period of the non-noise condition, indicating greater plasticity-like enhancement. These findings suggest that visual noise augments stimulus-induced VEP plasticity compared with the conventional non-noise condition, and that the SR mechanism may be critical for inducing plasticity-like modulation (Cooke and Bliss, 2006). It is also worth noting that the potentiation effect from early to late post-tetanus periods was limited to one component (N1) of the potentiated evoked response under the noise-tagged BCI intervention. The selective potentiation of only the N1 component following tetanization makes the possibility of overall changes of brain excitability unlikely, suggesting that the effect is instead due to an LTP-like process (Teyler et al., 2005).

Activation of synaptic transmission efficacy by task execution would result in brain excitability and corresponding response changes in specific cortical networks, and these changes are correlated with cognitive plasticity at the behavioral level (Fertonani et al., 2011; Smallwood et al., 2015). It should be noted that the rapid-frequency stimulation with visual noise used here to induce LTP in the visual cortex could parallel the change in visual performance. There is encouraging indication that visual perceptual discrimination thresholds in humans may be lowered after visual LTP induction (Beste et al., 2011; Çavuş et al., 2012; Montey et al., 2013). Therefore, once potentiated, these neuronal assemblies may be more likely to respond to previous subthreshold stimuli, which could allow people to see what they previously could not see (Clapp et al., 2012). For example, LTP has been inferred as the basis for the after-practice increase of visual contrast sensitivity in adults, and it was verified that with the utilization of noise, the performance of visual texture discrimination may be improved due to the activity-dependent plasticity (Simonotto et al., 1997). Therefore, the visual noise-enhanced potentiation would be a potential approach to study the improvement of visual performance.

Together, our results demonstrate that the enhancement of the response potentiation of VEPs can be induced by visual noise and measured in the human visual cortex. The ability to manipulate cortical plasticity-like potentiation with noninvasive rapid and repetitive sensory stimuli via the SR formalism should allow the detailed examination of sensory-induced plasticity in healthy humans. Larger frequency range with more tetanic-stimulation frequencies could be involved in the future work for further sophisticated studies of the brain SR influence on the potentiation of LTP-like plasticity. Moreover, it might also offer both rehabilitative and therapeutic strategies for facilitating the recovery from visual deficits, such as agnosia, amblyopia, and other detection-deficiency diseases, in future clinical applications (Maya Vetencourt et al., 2008; Montey et al., 2013; Casco et al., 2014). Conversely, the knowledge of brain plasticity could be used to design specific sensory stimulation protocols for the changes in brain organization and thus in perception and behavior; one might also integrate repetitive sensory stimulation and visual noise with motor training or other neuro-rehabilitative interventions to boost their functional impact and produce synergistic effects in practical applications (Cattaneo et al., 2010; Raffin and Siebner, 2014).

## Author contributions

JX conceived of the study, participated in the design of the study, carried out the experiments, and wrote the manuscript. GX and XZ designed the study. ML carried out the experiments and corrected the language. JW carried out the statistical analyses. CH collected field data. XH participated in data analysis. All authors agree to be accountable for the content of the work.

### Conflict of interest statement

The authors declare that the research was conducted in the absence of any commercial or financial relationships that could be construed as a potential conflict of interest.
